# The American Medical Association and Medical Colleges

**Published:** 1849-05

**Authors:** 


*The American Medical Association and Medical Colleges.*—The last No. of the New York Annalist, contains a series of resolutions, recently adopted by the New York Academy of Medicine, the burthen of which is the old story of rendering the degree of Doctor in medicine nugatory as a licence to practice. The Academy earnestly solicits the American medical Association to bring "the subject before the Legislatures of the several States, to procure the passage of such enactments as shall produce this important result."

The Editor of the Annalist states, that "the object sought for by these resolutions is well known to be the identical one that first gave rise to those movements in this state which resulted in the call for, and the final successful organization of the National Association." We presume this may be the case, and the editor of the Annalist is doubtless a competent witness on the subject, in as much as he was the one to move at the meeting of the N. Y. State Society a resolution for the call for a Convention, and was Chairman of the Committee appointed to carry the resolution into effect, of which fact the medical public have not been allowed to remain unreminded. But granting all this, we are at a loss to see why the motives and ends of the mover of the resolution, and some others, should furnish rules for the guidance of the Convention, and Association, resulting as a consequence of the action of the New York State Society. This matter referred to was brought up at the first Convention, in New York, and a committee, after considerable discussion, appointed to report especialy upon it. Two reports were submitted by the Committee, and acted upon at the meeting of the Association the following year at Philadelphia. The members of the Association at this meeting did not see fit to concur in the wishes of those who, for this single object, had labored to bring the Association into existence. Whether the subject was again discussed at the meeting at Baltimore, the following year, we are unable to say, without consulting the transactions. Yet, certain it is, that the Association did not at that time deem it expedient to gratify the few gentlemen who had engaged in the enterprise with this one idea. These successive failures in a predetermined plan, however, do not appear to discourage other efforts; and now it is announced the New York Academy of Medicine has been persuaded to move in the matter, in order to bring the subject again before the Association under better auspices.

The merits of this project we do not intend to discuss at any length. We deem it unnecessary to do so. Its impracticability, uselessness, and absurdity, have again and again, been demonstrated. It requires, in the first place, an united legislative action by the several States, which could never be attained, and which, it is generally supposed, if procured, would be unconstitutional, from its conflicting with rights already vested in corporate institutions. Putting all this out of question, what reasons can be assigned for supposing that a board of examiners composed of medical men not engaged in teaching, would be more competent to decide on qualifications for practice, or would be more strict in their requisitions, than a board consisting of, or, at least, embracing, professors in a Medical College? On the other hand, are there not some circumstances which should give teachers an advantage in estimating the abilities and attainments of medical pupils? They have been associated for months, and thus have had ample opportunities to become intimately acquainted with their characters, habits, and attainments. They are, it is to be presumed, more familiar, each with the details of the particular department which it is his province to teach, so that they should be better prepared to conduct a private and thorough examination. The responsibility to the medical profession is certainly as great in the one case as in the other, and in the case of those connected with Medical Schools, there are additional responsibilities arising from the fact that the character of the Institution is in no small measure involved in the character of its Alumni. We have never heard any reason assigned in favor of the proposed change, except the paltry insinuations, that teachers may deem it an object to induce students to attend at institutions with which they are connected, by holding out the prospect of a diploma on easy terms; and that the graduation fee is, virtually, a pecuniary bribe. The latter insinuation is easily disposed of, since, so far as our knowledge extends, the Professors of Medical Schools have no participation in the revenue from graduation fees; and the former like the latter, in our humble estimation is an insinuation unworthy to spring from a generous mind. Do the advocates of this proposed measure of reform really regard the Medical teachers of this country as so meanly selfish, corrupt, and dishonest a portion of the profession, as to merit such an imputation! We know not that any peculiar prerogatives belong to the position of a Medical Teacher beyond the addition of not a small amount of toil, and anxiety, with, generally, an inconsiderable amount of profit; but it does seem that the office should not disannul claims upon brethren of the same profession for ordinary respect and courtesy, or, to say the least, the exercise of common charity. The insulting nature of the suspicions so freely uttered by some restless spirits, appears to be quite lost sight of, from the frequent indulgence of opprobrious charges, and the eagerness to accomplish the desired ends. And what are these ends? Do they relate, purely and exclusively, to the improvement of Medical education? If so, why not attempt to make Medical Institutions better, if they are defective! Why not investigate malversations and corruption, if they are believed to exist, and endeavor to reform existing Institutions, and elevate them to be all that could be desired! This would be assuredly as effectual a remedy for existing evils (assuming that evils do exist) and far more practicable than the scheme which arises to depreciate and embarrass Medical Colleges.

Whatever may have been the intentions which prompted the movement resulting in the formation of the American Medical Association, we trust that the Association, as formed, will continue to repudiate all factious and disorganizing plans. United zeal and action for the promotion of legitimate and proper ends, will render the Association invaluable to the medical science of this country, and highly conducive to the honor and best interests of the profession, but if it be permitted to become the arena of intrigue, discord and strife, it requires no seer to predict its destiny.

